# Response to Letter to the Editor concerning Schmedding et al.: Decentralised surgery of abdominal wall defects in Germany (Pediatr Surg Int (2020) 36: 569–578)

**DOI:** 10.1007/s00383-020-04718-9

**Published:** 2020-07-22

**Authors:** Andrea Schmedding

**Affiliations:** Department of Pediatric Surgery and Pediatric Urology, University Hospital, Goethe University Frankfurt, Theodor‑Stern‑Kai 7, 60590 Frankfurt, Germany

Dear Sir,

We thank Dr. Schmiedecke and co-workers for commenting on our paper.

Our study utilized insurance data of patients with abdominal wall defects and identified mortality beside the length of hospital stay as one of the main outcome measures. Mortality is easy to assess in insurance data and is, therefore, an unbiased outcome parameter in this kind of studies. Somatic and psychosocial follow-up is impossible to evaluate using the provided insurance data, since they only cover 12 months after discharge from the first in-hospital stay.

We are appreciative of your comment on the missing table or graph on the case volume—mortality relation. Unfortunately, our study included specific insurance data, with no data of case numbers per treating institution due to data protection policy.

Our previous published study revealed that abdominal wall defects were treated in 86 paediatric surgical units with an average case load of less than five cases per institution [1]. Further, we have utilized the “Quality Reports of the Hospitals” (Gemeinsamer Bundesausschuss—GBA). The analysis of the Quality Reports revealed a maximum average case load of 12 procedures in only one institution for the period 2012–2015. No institution performed more than seven procedures for closure of abdominal wall defects in each of the specified years [2]. With these data, no high-volume centre with an average of more than 15 cases per year could be identified for Germany.

Youssef et al. reported on higher mortality rates in complex gastroschisis in the US [3]. Our data confirmed a higher mortality of complex gastroschisis which corresponds with many other studies. We do agree with the statement that the current situation in Germany compares with that of the US.

Hong et al. and Dubrovsky et al. published that higher centre volumes were not associated with improved outcome [4, 5]. This again is consistent with our findings. Sømme et al. investigated esophageal atresia, CDH and gastroschisis and did not find an association between volume and outcome [6].

The statement that paediatric surgeons oppose centralization in general is invalid, since it is not supported by available data. We completely agree to centralize complex paediatric surgical care. Furthermore, we call for a national registry for congenital anomalies as a basis for follow-up evaluation and quality management [7].

Sincerely

Andrea Schmedding.
